# A Low-Cost Fertilizer Medium Supplemented with Urea for the Lutein Production of *Chlorella* sp. and the Ability of the Lutein to Protect Cells against Blue Light Irradiation

**DOI:** 10.3390/bioengineering10050594

**Published:** 2023-05-15

**Authors:** Chiu-Mei Kuo, Yi-Chun Yang, Wen-Xin Zhang, Jia-Xun Wu, Yu-Tso Chen, Cheng-Han Lin, Meng-Wei Lin, Chih-Sheng Lin

**Affiliations:** 1 Department of Chemical Engineering, Chung Yuan Christian University, Taoyuan City 320314, Taiwan; cmkuo@cycu.edu.tw (C.-M.K.); philip880916@gmail.com (J.-X.W.); qq34887088@gmail.com (Y.-T.C.); 2 Department of Biological Science and Technology, National Chiao Tung University, Hsinchu 30068, Taiwan; dorothy324047@gmail.com (Y.-C.Y.); patrickh0518@gmail.com (W.-X.Z.); 3 Department of Biological Science and Technology, National Yang Ming Chiao Tung University, Hsinchu 30068, Taiwan; a0975273923@gmail.com (C.-H.L.); lmw1018@nycu.edu.tw (M.-W.L.)

**Keywords:** *Chlorella*, fertilizer, urea, lutein, blue light, ROS

## Abstract

This study aimed to investigate the use of organic fertilizers instead of modified f/2 medium for *Chlorella* sp. cultivation, and the extracted lutein of the microalga to protect mammal cells against blue-light irradiation. The biomass productivity and lutein content of *Chlorella* sp. cultured in 20 g/L fertilizer medium for 6 days were 1.04 g/L/d and 4.41 mg/g, respectively. These values are approximately 1.3- and 1.4-fold higher than those achieved with the modified f/2 medium, respectively. The cost of medium per gram of microalgal biomass reduced by about 97%. The microalgal lutein content was further increased to 6.03 mg/g in 20 g/L fertilizer medium when supplemented with 20 mM urea, and the cost of medium per gram lutein reduced by about 96%. When doses of ≥1 μM microalgal lutein were used to protect mammal NIH/3T3 cells, there was a significant reduction in the levels of reactive oxygen species (ROS) produced by the cells in the following blue-light irradiation treatments. The results show that microalgal lutein produced by fertilizers with urea supplements has the potential to develop anti-blue-light oxidation products and reduce the economic challenges of microalgal biomass applied to carbon biofixation and biofuel production.

## 1. Introduction

The 26th United Nations Climate Change Conference of the Parties in 2021 called for urgent climate action to halve global greenhouse gas emissions by 2030 and reach net zero by 2050. Photosynthetic carbon dioxide (CO_2_) biofixation by microalgae is a promising technology for carbon fixation because a microalgae culture has higher growth rates, lower water requirements, and occupies a smaller land area than terrestrial plants [1]. The resulting microalgal biomass can be used to produce lipids and carbohydrates as a source of chemical precursors and biofuels [2,3]. It is also possible to produce key bioactive compounds, such as astaxanthin, β-carotene, canthaxanthin, chlorophylls, lutein, proteins, and vitamins. These key bioactive compounds can be applied to high-value products such as feed, cosmeceuticals, pharmaceuticals, nutraceuticals, and food supplements through biorefinery [4,5].

Circular bioeconomy of microalgae-based CO_2_ biofixation and biorefinery can be analyzed through technical–economic assessment (TEA) and life-cycle assessment (LCA) [4,5,6]. However, one of the bottlenecks faced by microalgae-based CO_2_ biofixation technology is the high cost of microalgal culture medium; the components in the culture medium are among the key factors that greatly affect the growth of microalgae and the production of various components [7,8]. Nutrient requirements in large-scale production can comprise up to 50% of the total cost of biomass production, and high-cost media limit the development of back-end products such as biofuels [9,10,11]. Therefore, many studies have used nutrients in wastewater, including chemical oxygen demand (COD), total nitrogen (TN), total phosphorus (TP), and specific inorganic substances, reutilizing them in microalgae cultivation to reduce the cost of culture medium [12,13,14,15,16,17]. However, the microalgal biomass generated by reusing wastewater limits the application of microalgae-based products due to biosafety considerations. Furthermore, changes in the macro- and micronutrient content of wastewater, heavy metal contamination, and the presence of competitors and pathogens can lead to unstable or even reduced microalgal biomass production [18]. The production of microalgal biomass by aquaculture wastewater would be less prevalent than the biosafety considerations because the components of aquaculture wastewater are simpler and because it contains fewer pathogenic microorganisms and low amounts of heavy metals [19,20]. *Scenedesmus almeriensis* CCAP 276/24, cultured with fertilizer media in raceway, had a significantly lower presence of pathogens than that with municipal wastewater [21].

Some studies have also investigated fertilizers and additional supplementary compounds as low-cost potential media for microalgae cultivation. *Ankistrodesmus gracile* was cultured in the mixture of macrophyte *Eichhornia crassipes* extract and inorganic fertilizer, and the microalgal growth rate and lipid content was enhanced compared with that of synthesized CHU12 medium [22]. When organic liquid fertilizer PAL-1 was used as the culture medium of the microalga *Chlorella vulgaris*, the microalgal lipid content was increased to 48% [23]. *Scenedesmus obliquus* BR003 was cultured in alternative fertilizer-based media with ammonium and urea supplement to increase the microalgal lipid content production up to 25% dry weight, compared to BG11 medium [24]. A mixture of mono-ammonium phosphate, triple superphosphate, phosphoric acid, and ammonium nitrate fertilizers was used in the cultivation of *Dunaliella tertiolecta* MS029, and the amount of polyunsaturated fatty acids of microalgal lipid after transesterification was reduced to improve the quality of biodiesel [25]. *Chlorella sorokiniana* was cultivated in the combined use of urea, ammonia, and nitrate-based fertilizers with an increase of 7% protein, 41% carotenoids, 12% soluble sugar, 370% alanine, 350% serine, 180% valine, 190% myo-inositol, 230% glyceric acid, and 220% glutamic acid, compared to BG11 medium [26]. The lipid of *Tetradesmus obliquus* cultured in farm fertilizers with optimal urea supplementation was 127.1 mg/L, equivalent to that in N11 medium [27]. The maximum lipid content of *Scenedesmus* sp. IMMTCC-6 cultured in the inorganic fertilizer and urea as medium was about 28.6%, compared to the control medium which contained urea only [28]. When commercial fertilizers were used instead of TAP medium, the growth and hydrogen production of *Chlamydomonas reinhardtii* CC124 performed well [29]. These studies demonstrated that fertilizers can be used as culture media for many different microalgal species, replacing synthetic media to reduce costs, and potentially realize the benefits of increased microalgal biomass production and production of specific metabolites such as lutein, β-carotene, and zeaxanthin.

Lutein is one of the main pigments in microalgae and higher plants. As an accessory pigment, lutein can participate in light harvesting and light protection, as well as energy transfer, and is characterized by blue-light filtering and antioxidant properties [30]. Exposing cells to blue light causes oxidative damage to cell membranes and damages the mitochondria, increasing intracellular reactive oxygen species (ROS) concentrations [31,32]. Removal of lutein from the diet of experimental animals resulted in early signs of retinal degeneration, as lutein accumulates in the lens and macula of the retina and protects the retina from light-induced oxidative stress [33,34]. According to the 2023 Future Market Insights market report, the global lutein market was estimated to be USD 354 million in 2022, and it is expected to reach USD 590 million by 2032; the compound annual growth rate in 2022–2032 is expected to reach 5.2% [35]. Microalgae is a potential source of lutein that is very competitive in the commercial market. At present, the main commercial source of lutein is marigold flowers [36,37]. Compared with marigold flowers, microalgae have the advantages of a fast growth rate, wide adaptability to growth conditions, and can be harvested all year round. The productivity of microalgal lutein was three–six times higher, and the highest annual output can reach 350~750 kg/ha [38,39]. Many studies have reported producing lutein using *Chlorella* species, including *Chlorella minutissima* [40], *Chlorella sorokiniana* MB-1-M12 [41], *Chlorella* sp. AE10 [42], *Chlorella sorokiniana* FZU60 [43,44], *Chlorella sorokiniana* F31 [45], and *Chlorella sorokiniana* Kh12 [46]. It can be seen that the use of *Chlorella* sp. to produce lutein raw materials with high market demand can be used as a potential strategy to reduce the economic challenges of CO_2_ biofixation by microalgae. In this study, organic fertilizers were used to replace the chemically synthesized medium in *Chlorella* sp. cultivation, and additional components were added to increase the content of lutein to construct a *Chlorella* sp. culture process using the low-cost medium. The produced lutein of *Chlorella* sp. was evaluated for the ability of NIH/3T3 cell protection to reduce blue-light-induced ROS production.

## 2. Materials and Methods

### 2.1. Microalgal Cultures, Medium, and Chemicals

The *Chlorella* sp. was originally obtained from Taiwan Fisheries Research Institute (Tung-Kang, Taiwan), and screened for high-growth ability at National Yang Ming Chiao Tung University, Taiwan [20]. The modified f/2 medium was used for the cultivation of *Chlorella* sp. and was composed of (per liter): 29.23 g NaCl, 1.105 g KCl, 11.09 g MgSO_4_·7H_2_O, 1.21 g Tris-base, 1.83 g CaCl_2_·2H_2_O, 0.25 g NaHCO_3_, and 3 mL of trace-metal solution. The trace-metal solution was composed of (per liter): 75 g NaNO_3_, 5 g NaH_2_PO_4_·H_2_O, 4.36 g EDTA·2Na, 3.16 g FeCl_3_·6H_2_O, 180 mg MnCl_2_·4H_2_O, 10 mg CoCl_2_·6H_2_O, 10 mg CuSO_4_·5H_2_O, 23 mg ZnSO_4_·7H_2_O, 6 mg Na_2_MoO_4_, 100 mg vitamin B1, 0.5 mg vitamin B12, and 0.5 mg biotin.

### 2.2. Preparation of the Inoculum

The stock culture of *Chlorella* sp. was incubated in a column-type photobioreactor containing 1 L working volume of the modified f/2 medium with 2% CO_2_ at an aeration rate of 0.2 vvm (volume of gas per volume of medium per min), 26 ± 1 °C and 300 μmol/m^2^/s. The logarithmic phase of *Chlorella* sp. was used as an inoculum by dilution to obtain the initial biomass concentration of 0.3 g/L for further experiments.

### 2.3. Batch Cultivations of Indoor Photobioreactors

The column-type glass-fabricated photobioreactors (*ϕ* 6 cm × 80 cm high) were used for the microalgal cultures with 1 L of working volume [47]. The microalgal cells were cultured at 26 ± 1 °C and were continuously illuminated at 300 μmol/m^2^/s intensity of cool-white fluorescent lights on the photobioreactor’s surface. The gas containing 2% CO_2_ was premixed with air and supplied from the bottom of the photobioreactor with an aeration rate of 0.2 vvm. The initial biomass concentration of the *Chlorella* sp. was approximately 0.3 g/L. The microalgal cells in each culture were sampled at 24 h intervals to determine the biomass concentration.

To evaluate the growth and lutein content of *Chlorella* sp., the microalgal cells were cultured with 10, 20, and 30 g/L solid organic fertilizer as the culture medium, and the modified f/2 medium. According to our study [48], the organic fertilizer used in this study contained 24% TN, 5.1% water-soluble phosphoric anhydride, and 20.5% water-soluble potassium oxide.

To enhance the growth and lutein content of *Chlorella* sp., the microalgal cells were cultured in 20 g/L fertilizer by adding 3 nitrogen-based compounds individually for 7 days, including 0, 25, 50, 100, and 200 μM nicotine, 0, 5, 10, 20, 30, and 60 mM NaNO_3_, and 0, 5, 10, 20, 30, and 60 mM urea.

### 2.4. Determination of Microalgal Cell Biomass and Biomass Productivity

According to the method used in our previous study [49], a calibration equation of microalgal biomass concentration (dry weight per liter) and optical density was established, where the optical density was measured at a wavelength of 682 nm (A_682nm_); its absorbance range was between 0.1 and 1, as shown in Equation (1).

Biomass concentration (g/L) = 0.3094 × A_682nm_ + 0.003 (1)

The microalgal biomass concentration could be accurately calculated by detecting A_682nm_ (R^2^ = 0.9952; *p* < 0.001). When the optical density A_682 nm_ exceeded 1, the sample of microalgae needed to be diluted sufficiently before measurement. Biomass productivity (g/L/d) was calculated using Equation (2):

Biomass productivity (g/L/d) = (W_2_ − W_1_)/(t_2_ − t_1_) (2)

where W_2_ and W_1_ are the biomass concentrations (g/L) at t_2_ and t_1_ (day), respectively.

### 2.5. Determination of Microalgal Lutein Content

Lutein was extracted using a method modified from that of Xie et al. [50]. The lutein content of the extract was analyzed using 1260 Infinity II high-performance liquid chromatography (Agilent, Santa Clara, CA, USA), as proposed by Taucher et al. [51]. A reversed-phase YMC carotenoid C30 column (150 × 4.6 mm, 3 μm) operated at 22 °C with mobile phase 1 mL/min. The mobile phase consists of two solvent mixtures: (A solvent) methanol, methyl tert-butyl ether (MTBE), and water: 81:15:4; and (B solvent) methanol, MTBE, and water: 8:89:3 (v:v:v). The mobile phase is a gradient condition, first flowing at 98% (A solvent) for 11 min, then decreasing to 60% (A solvent) over the next 7 min. This is maintained for 6.5 min, then decreases to 0% (A solvent) in 2.5 min and held for 3 min; it then increases to 98% (A solvent) over the next 3 min to re-equilibrate the mobile phase and is held for 7 min. Lutein was detected by measuring the absorbance at the wavelength 445 nm. The lutein standard was purchased from Sigma Chemical Co. (St. Louis, MO, USA). The lutein content (mg/g) and lutein productivity (mg/L/d) of microalgae were calculated as shown in Equations (3) and (4).

Lutein content (mg/g) = Lutein concentration (mg/L)/Biomass concentration (g/L)(3)

Lutein productivity (mg/L/d) = Lutein content (mg/g)/Biomass productivity (g/L/d)(4)

### 2.6. Cell Culture and Medium

NIH/3T3 Swiss albino mouse fibroblast cells BCRC 60071 were purchased from Bioresource Collection and Research Center (Hsinchu, Taiwan). NIH/3T3 cells were cultured in Dulbecco’s modified Eagle’s medium (DMEM) with 10% bovine calf serum and maintained in a humidified atmosphere of 5% CO_2_ at 37 °C. The cells were washed with Dulbecco’s phosphate-buffered saline (DPBS) and detached from the culture surface using 0.25% trypsin-EDTA solution to subculture and seed.

### 2.7. Evaluation of the Ability of Microalgal Lutein to Protect Cells against Blue-Light Irradiation

Another study tested the ability of lutein and astaxanthin to protect human corneal epithelial cells from blue-violet light photo-oxidative damage [34]. First, to investigate the cell viability and accumulation of ROS by blue-light irradiation with 300 μmol/m^2^/s intensity, the NIH/3T3 cells were exposed to blue light for 0, 3, 6, 12, 18, and 24 h. The NIH/3T3 cells without blue-light irradiation (0 h) were defined as the control.

Next, to evaluate the protective effect of microalgal lutein on the cells against blue-light irradiation, NIH/3T3 cells were incubated with 10 μM lutein supplement for 0, 1, 3, 6, 12, and 24 h of pre-treated time, and then irradiated with a 300 μmol/m^2^/s intensity of blue light for 12 h to measure the ROS produced by the cells. To further investigate the dose–effect response of microalgal lutein to protect the cells damaged by blue-light irradiation, NIH/3T3 cells were incubated with 0, 0.01, 0.1, 1, and 10 μM lutein for a pre-treatment time of 6 h, and then irradiated at a 300 μmol/m^2^/s intensity of blue light for 12 h to measure the ROS produced by the cells. The NIH/3T3 cells without microalgal lutein supplement and without blue-light irradiation were defined as the control.

### 2.8. Determination of Cell Viability

The 3-(4,5-dimethylthiazol-2-yl)-2,5-diphenyl tetrazolium bromide (MTT) assay has been recognized as a standard method for determining cell viability [52]. The effect of microalgal lutein on cell viability used the method based on that of Gong et al., 2017 [53]. The NIH/3T3 cells were seeded in a 24-well plate (5 × 10^4^ cells/well) and incubated in humidified 5% CO_2_ at 37 °C for 24 h prior to the ability of microalgal lutein evaluation. The microalgal lutein was dissolved in sterile dimethyl sulfoxide (DMSO). The cells were treated with microalgal lutein for a predetermined testing period. Subsequently, 5 mg/mL of MTT solution was added to each well for 4 h incubation to aspirate supernatant in wells and DMSO was added to dissolve the purple formazan crystals. Finally, the absorbance of produced formazan solution was detected at A570 nm. The results were expressed as the cell viability % of the control group.

### 2.9. Determination of ROS Production

ROS production was detected using a non-fluorescent compound 2′,7′-dichlorodihydrofluorescein diacetate (DCFH-DA) to produce impermeable 2′,7′-dichlorofluorescin (DCFH) via esterase hydrolysis through passive diffusion into cells. DCFH was oxidized to fluorescent dichlorofluorescein (DCF) by ROS [54]. First, NIH/3T3 cells were treated with 20 μM DCFH-DA for 30 min at 37 °C in darkness. Then, the cells were washed twice with phosphate-buffered saline, and the fluorescence intensity of DCF in the NIH/3T3 cells was measured using a fluorescence spectrophotometer F-2700 (Hitachi High-Tech., Pleasanton, CA, USA) with excitation and emission wavelengths at 488 nm and 525 nm.

### 2.10. Statistics

The values are expressed as the mean ± standard deviation (SD). Three replicates were tested for each measurement. Data were compared using a one-way analysis of variance (ANOVA) test to evaluate the differences between multiple groups. The differences were considered statistically significant using different letters when *p* < 0.05. Statistical significance between the two groups was evaluated using the two-tailed paired Student’s t-test; the differences were indicated with asterisks (* *p* < 0.05, ** *p* < 0.01, and *** *p* < 0.001). Statistical analysis was performed using statistical software (SPSS, Chicago, IL, USA).

## 3. Results and Discussion

### 3.1. Chlorella sp. Was Cultivated in Fertilizer

**Figure 1:**
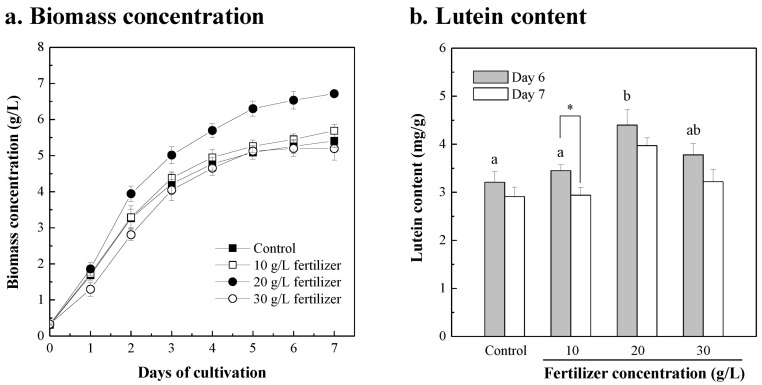
Growth profiles (**a**) and lutein contents (**b**) of the *Chlorella* sp. cultured in different fertilizer concentrations (10, 20, and 30 g/L), and compared to the cultures in modified f/2 medium as control. The initial microalgal biomass concentration was approximately 0.3 g/L. The culture was operated at 26 ± 1 °C with 300 μmol/m^2^/s of light intensity and with a 2% CO_2_ aeration rate of 0.2 vvm for 7 days. The microalgal cells were sampled every 24 h for growth determinations and in a 6-day and a 7-day culture for lutein content determinations. Data were compared with a one-way ANOVA test to evaluate the differences between multiple groups on day 6. Different letters indicate significant differences between groups (*p* < 0.05). Statistical significance for each group on day 6 and day 7 is indicated by asterisks. Two-tailed paired Student *t*-test *p*-values indicate statistical significance (* *p* < 0.05).

To reduce production costs in microalgal cultivation, the *Chlorella* sp. was cultivated in different fertilizer concentrations, with the cultures in modified f/2 medium as the control group. As shown in Figure 1a, the maximum biomass concentrations of *Chlorella* sp. cultivation in 10, 20, and 30 g/L fertilizer for 7 days were 5.69, 6.72, and 5.19 g/L, and the biomass productivity for 7 days was 0.769, 0.914 and 0.695 g/L/d, respectively. The resulting biomass of *Chlorella* sp. on day 6 and day 7 was extracted for lutein analysis; the microalgal lutein content at day 6 in each group was higher than that on day 7 (Figure 1b). The maximum lutein content and lutein productivity of *Chlorella* sp. Cultivation in 10, 20, and 30 g/L fertilizer for 6 days was 3.45, 4.41, and 3.78 mg/g dry microalgal biomass, and 2.96, 4.56, and 3.06 mg/L/d, respectively. Compared with the control group of modified f/2 medium, the biomass productivity and lutein productivity of *Chlorella* sp. for 6 days were significantly enhanced by 1.3-fold to 1.04 g/L/day (*p* < 0.001) and 1.7-fold to 4.56 mg/L/day (*p* < 0.001) using 20 g/L fertilizer as the replacement culture medium. The results show that the fertilizer can replace the synthetic f/2 medium for microalgae cultivation, and 20 g/L was the optimal culture concentration. When 20 g/L fertilizer replaced modified f/2 medium, the microalgal biomass productivity and content of the high-value component lutein significantly increased and the cost decreased from USD 0.052 to USD 0.002 per gram of microalgal biomass, achieving a cost reduction of nearly 97%, and from USD 16.35 to USD 0.382 per gram of microalgal lutein, achieving a cost reduction of nearly 98% (Table 1). To improve the economic challenge of producing functional compounds in microalgae cultivation, some studies have also used fertilizers to replace chemically synthesized media to reduce the cost of media. Compared with the commercial ALGAL medium, when using agricultural fertilizer to cultivate *Isochrysis galbana* the microalgal biomass productivity, protein productivity, and docosahexaenoic acid (DHA) productivity were increased by 1.2-fold to 0.44 g/L/d, 1.3-fold to 197 mg/L/d, and 1.5-fold to 6.5 mg/L/d, respectively. The cost of the culture medium was reduced by 95% when agricultural fertilizer was used [55]. When *Chlorella vulgaris* was grown in aquaculture wastewater supplemented with commercial fertilizer NPK than Bold’s Basal medium, the microalgal biomass productivity and protein productivity were enhanced by 1.5-fold to 0.17 g/L/d, and 1.8-fold to 53 mg/L/d, respectively. The medium costs reduced by up to 99% due to the use of fertilizer and wastewater [11]. The abovementioned studies show that reducing the cost of media can reduce the economic challenges of large-scale microalgae cultivation and increase the opportunities for CO_2_ biofixation applications and biofuel development, which can also be accompanied by the development of high-value components, such as lutein, DHA, protein, etc.

### 3.2. Chlorella sp. Was Cultured in Fertilizer with the Nicotine Supplement

**Figure 2:**
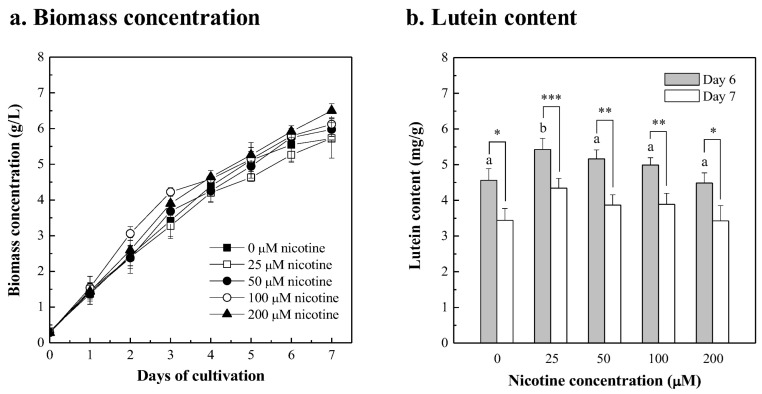
Growth profiles (**a**) and lutein contents (**b**) of the *Chlorella* sp. cultured in 20 g/L fertilizer with the supplement of different nicotine concentrations (25, 50, 100, and 200 μM), and compared to the cultures without nicotine supplement. The initial microalgal biomass concentration was approximately 0.3 g/L. The culture was operated at 26 ± 1 °C with 300 μmol/m^2^/s of light intensity and with a 2% CO_2_ aeration rate of 0.2 vvm for 7 days. The microalgal cells were sampled every 24 h for growth determinations and in a 6-day and a 7-day culture for lutein content determinations. Data were compared with a one-way ANOVA test to evaluate the differences between multiple groups on day 6. Different letters indicate significant differences between groups (*p* < 0.05). Statistical significance for each group on day 6 and day 7 is indicated by asterisks. Two-tailed paired Student *t*-test *p*-values indicate statistical significance (* *p* < 0.05, ** *p* < 0.01 and *** *p* < 0.001).

The carotenoid pathway was altered to form specific products in microalgae by adding chemical inhibitors, including nicotine, imidazole, piperidine, triethylamine, pyridine, and niacin [37]. Enhancement of the lutein content of *Chlorella* sp. by adding different concentrations of the chemical inhibitor nicotine in 20 g/L fertilizer media was also investigated. As shown in Figure 2a, the growth curve of *Chlorella* sp. was similar when a 25~100 μM nicotine supplement was added, compared to the control cultures without nicotine. Compared with the control group, the biomass concentration of *Chlorella* sp. significantly increased after 7 days of 200 μM nicotine supplementation (*p* < 0.01). The maximum biomass concentration of *Chlorella* sp. increased by about 1.2-fold to 6.5 g/L in 200 μM nicotine supplement for 7 days. The biomass productivity of *Chlorella* sp. cultured in 20 g/L fertilizer media with 0, 25, 50, 100, and 200 μM nicotine supplement for 7 days was 0.772, 0.774, 0.813, 0.833, and 0.888 g/L/day, respectively. The lutein content of *Chlorella* sp. at 6 days was higher than that at 7 days in each group (Figure 2b). Compared with the control group, the lutein content of *Chlorella* sp. was enhanced by adding 25~100 μM nicotine for 6 days, especially when 25 μM nicotine was added, due to the significant differences (*p* < 0.01). When *Chlorella* sp. was cultured in 20 g/L fertilizer media with 25 μM nicotine for 6 days, the maximum microalgal lutein content increased by about 1.2-fold to 5.4 mg/g dry weight, compared with the control group. This result shows that the lutein content in the carotenoid biosynthetic pathway of *Chlorella* sp. was enhanced by the addition of the chemical inhibitor nicotine. It is known that in the microalgal carotenoid biosynthetic pathway, lycopene cyclization is converted into β-carotene branch through lycopene β-cyclase (LCY-b), and into the α-carotene branch through lycopene ε-cyclase (LCY-e), respectively. The microalgal α-carotene branch was followed by lutein synthesis [56]. The lutein content of *Dunaliella salina* was increased 1.7-fold (3.455 pg/cell) when the chemical inhibitor 2-methylimidazole was added to mediate the carotenoid biosynthesis pathway, while the β-carotene content decreased [57]. Similar results show that adding nicotine to the carotenoid biosynthetic pathway of *Chlorella regularis* Y-21 or *Dunaliella salina* CCAP 19/18 achieved a reduction in the β-carotene content, and a significant increase in the content of lycopene and carotenoid intermediates [58].

### 3.3. Chlorella sp. Was Cultured in Fertilizer with NaNO_3_ Supplement

**Figure 3:**
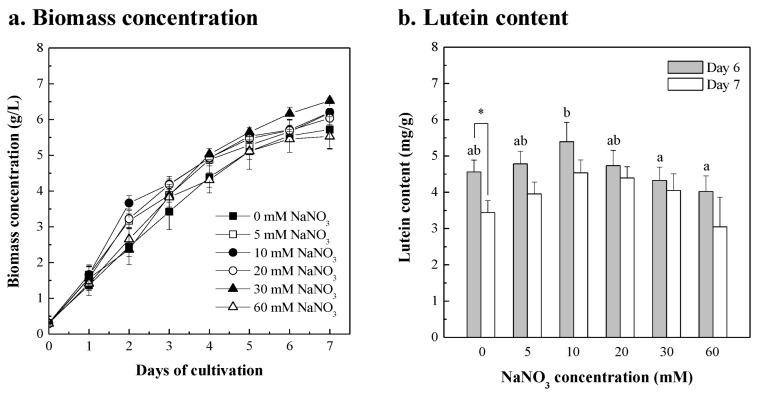
Growth profiles (**a**) and lutein contents (**b**) of the *Chlorella* sp. cultured in 20 g/L fertilizer with the supplement of different NaNO_3_ concentrations (5, 10, 20, 30, and 60 mM), and compared to the cultures without NaNO_3_ supplement. The initial microalgal biomass concentration was approximately 0.3 g/L. The culture was operated at 26 ± 1 °C with 300 μmol/m^2^/s of light intensity and with a 2% CO_2_ aeration rate of 0.2 vvm for 7 days. The microalgal cells were sampled every 24 h for growth determinations and in a 6-day and a 7-day culture for lutein content determinations. Data were compared with a one-way ANOVA test to evaluate the differences between multiple groups on day 6. Different letters indicate significant differences between groups (*p* < 0.05). Statistical significance for each group on day 6 and day 7 is indicated by asterisks. Two-tailed paired Student *t*-test *p*-values indicate statistical significance (* *p* < 0.05).

Nitrogen is a major component of microalgae for nucleic acid, protein, and pigment synthesis, and nitrogen availability affects microalgal biomass and the production of specific carotenoid metabolites, such as accumulation of lutein, fucoxanthin violaxanthin, antheraxanthin, and zeaxanthin [59,60,61]. To investigate the effect of adding nitrogen NaNO_3_ on the growth and the lutein content, the *Chlorella* sp. was cultured in 20 g/L fertilizer with different NaNO_3_ concentrations supplemented. As shown in Figure 3a, the biomass productivity of *Chlorella* sp. cultured in 20 g/L fertilizer with 5, 10, 20, 30, and 60 mM NaNO_3_ for 7 days was 0.839, 0.842, 0.821, 0.890, and 0.748 g/L/day, respectively. Compared with the control group without NaNO_3_, the growth curve of *Chlorella* sp. with 5~60 mM NaNO_3_ supplementation was similar, and a higher biomass concentration was obtained by adding 30 mM NaNO_3_ for 7 days of cultivation. However, when a high concentration of 60 mM NaNO_3_ was added, the biomass concentration and lutein content of *Chlorella* sp. was lower compared to the control group. Similar results were also shown by Altın et al. [62]; the NaNO_3_ supplement in BG-11 medium did not significantly improve the growth rate of *Chlorella variabilisin*. However, nitrate supplementation aided the growth of *Scenedesmus obliquus* FSP-3, *Nannochloropsis oculata*, *Tetraselmis tetrathele*, and *Chlorella vulgaris* [63,64,65]. Regardless of whether NaNO_3_ was added or not, the lutein content of *Chlorella* sp. harvested at day 6 of culture was higher than at day 7, and the maximum lutein content was 5.4 mg/g with the addition of 10 mM NaNO_3_ (Figure 3b). The results show that the supplementation of 10 mM NaNO_3_ at an appropriate concentration was beneficial to the enhancement of lutein content, and had no inhibitory effect on the growth of *Chlorella* sp. The similar results revealed that the lutein content of *Muriellopsis* sp. showed no significant change with the supplementation of nitrogen sources NaNO_3_, NH_4_Cl, and NH_4_NO_3_ [66], and the lutein content of *Nephroselmis* sp. N3C46 was stable with sufficient nitrogen supplementation [61]. This means that the correlation between nitrogen source supplements, microalgal growth, and lutein content varies among microalgal species.

### 3.4. Chlorella sp. Was Cultured in Fertilizer with the Urea Supplement

**Figure 4:**
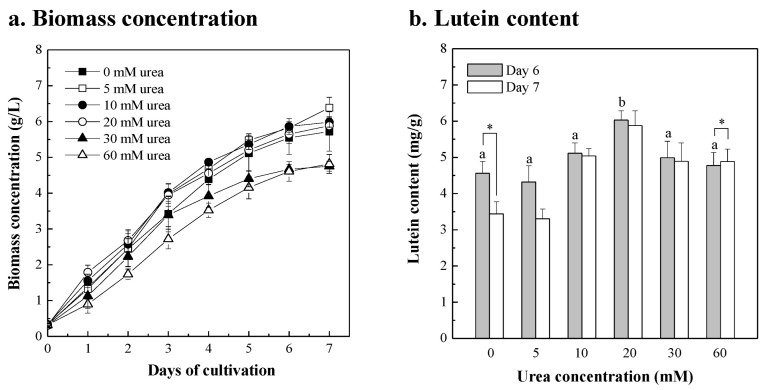
Growth profiles (**a**) and lutein contents (**b**) of the *Chlorella* sp. cultured in 20 g/L fertilizer with the supplement of different urea concentrations (5, 10, 20, 30, and 60 mM), and compared to the cultures without urea supplement. The initial microalgal biomass concentration was approximately 0.3 g/L. The culture was operated at 26 ± 1 °C with 300 μmol/m^2^/s of light intensity and with a 2% CO_2_ aeration rate of 0.2 vvm for 7 days. The microalgal cells were sampled every 24 h for growth determinations and in a 6-day and a 7-day culture for lutein content determinations. Data were compared with a one-way ANOVA test to evaluate the differences between multiple groups on day 6. Different letters indicate significant differences between groups (*p* < 0.05). Statistical significance for each group on day 6 and day 7 is indicated by asterisks. Two-tailed paired Student *t*-test *p*-values indicate statistical significance (* *p* < 0.05).

Carbon and nitrogen are essential to the growth of microalgae, and urea is a source of both carbon and nitrogen [67,68]. The growth and lutein content of *Chlorella* sp. in 20 g/L fertilizer with different urea concentrations was investigated. As shown in Figure 4a, the biomass productivity of *Chlorella* sp. cultured in 20 g/L fertilizer with 5, 10, 20, 30, and 60 mM urea for 7 days was 0.865, 0.807, 0.796, 0.634, and 0.642 g/L/day, respectively. Compared with the control group without added urea, the growth of *Chlorella* sp. was significantly inhibited when 30 mM and 60 mM urea were added (*p* < 0.01), while the microalgal growth curve when 5, 10, and 20 mM urea was added was similar to that of the control group. When a low concentration of urea was added, there was no significant difference in the concentration of microalgal biomass. Similar results were also shown in the report by Kim et al. [69]. *Chlorella* sp. HS2 was cultured in BG-11 medium supplemented with 2.1~12.5 mM urea; there was no correlation between microalgal biomass concentration and urea concentration. As for the lutein content, Figure 4b shows that the lutein content of *Chlorella* sp. with a urea supplement exhibited no inhibitory effect and the lutein content increased significantly following the addition of 20 mM urea supplement. During a 6-day cultivation period, the lutein content of *Chlorella* sp. with 20 mM urea supplement reached a maximum of 6.03 mg/g, an increase of 1.3-fold compared with the control group. The results show that urea supplementation mainly influenced the lutein content in *Chlorella* sp. in this study. For *Chlorella protothecoides* CS-41, the lutein content was increased to 4.58 mg/g by adding about 60 mM urea as a nitrogen source [70]. When *Chlorella sorokiniana* MB-1-M12 was cultured in BG-11 medium supplemented with 0.75 g/L urea in 250 mL flasks and 5 L fermenters, its maximum lutein content was 3.67 mg/g and 5.88 mg/g, respectively [41].

Since the maximum lutein content of *Chlorella* sp. can be obtained after 6 days of culture, based on the results of adding the extra components of nicotine, NaNO_3_, and urea to the 20 g/L fertilizer medium for 6 days, the data of groups with extra added components and the control group as 20 g/L fertilizer medium were compared with those obtained via a one-way ANOVA test to evaluate the significant differences on biomass concentration, biomass productivity, lutein content, and lutein productivity (Table 2). In terms of biomass concentration and biomass productivity, there was no significant difference between groups with an additional 25~200 μM nicotine, 5~60 mM NaNO_3_, 5~20 mM urea, and 20 g/L fertilizer without extra components of the control. However, the supplementation of high concentrations of 30 and 60 mM urea significantly inhibited the growth of *Chlorella* sp. compared with other groups. There was only a significant difference in the increase in lutein content and lutein productivity between the group with extra added 20 mM urea and the control group. The maximum lutein productivity of *Chlorella* sp. was 5.36 mg/L/day with the addition of 20 mM urea in 20 g/L fertilizer. In addition, urea is a widely used and cheap nutrient source for microalgal cultivation [71,72]. Therefore, when 20 mM urea was added to the 20 g/L fertilizer medium to replace the f/2 medium, the cost of media was reduced from USD 16.35 to USD 0.665 per gram lutein, a reduction of 96% (Table 1). Similar results were obtained for *Chlorella sorokiniana* MB-1-M12, in which the cost reduction was 84% when urea was used as the nitrogen source [41]. When urea, ammonia, and nitrate were added to the commercial fertilizer to replace the BG-11 medium, the medium’s cost of 1 kg biomass of *Chlorella sorokiniana* produced decreased by 95% [26]. Developing low-cost media helps reduce the economic challenges of developing products from microalgal biomass [27].

### 3.5. Damage of NIH/3T3 Cells from Blue-Light Irradiation

**Figure 5:**
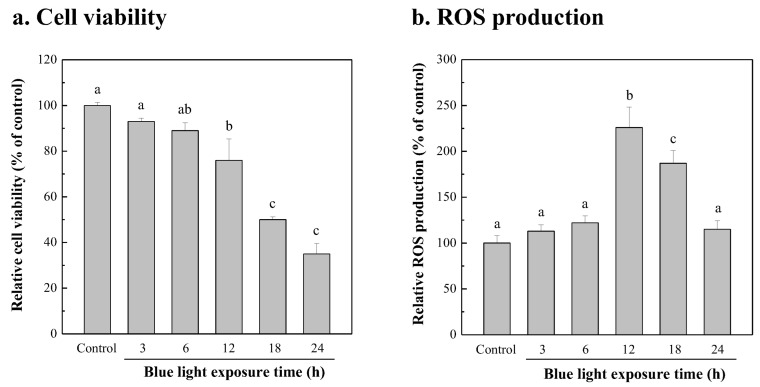
Cell viability (**a**) and ROS production (**b**) of NIH/3T3 cells treated by blue light for the different irradiated time. NIH/3T3 cells were exposed to blue light with 300 μmol/m^2^/s of intensity for 3, 6, 12, 18, and 24 h, cell viability was detected using an MTT assay and the ROS production. The cell viability and ROS production of NIH/3T3 cells without exposure to blue light (0 h) were defined as the control (C; 100%). All values were expressed as the mean ± SD. Data were compared with a one-way ANOVA test to evaluate the differences among multiple groups. Different letters indicate significant differences between groups.

ROS in cells are generated by blue-light irradiation [73,74]. Many studies have demonstrated that lutein can resist the damage of blue-light irradiation [34,75,76]. The raw materials of the lutein used are mainly extracted from plants [77,78] or produced in synthetic media by microorganisms [79,80] and microalgae [6,81]. In this study, the lutein produced by a biomass of *Chlorella* sp. cultured in 20 g/L fertilizer with a 20 mM urea supplement was used to investigate resistance to blue-light irradiated damage. Firstly, the NIH/3T3 cells were irradiated with blue light for different exposure times to investigate the relationship between the ROS content in the cells and the cell viability resulting from blue-light irradiation. The cell viability effect of blue light under different exposure times (3, 6, 12, 18, and 24 h) on NIH/3T3 cells is shown in Figure 5. Lower viability of NIH/3T3 cells concurred with a longer period of blue-light irradiation. As shown in Figure 5a, when the NIH/3T3 cells were exposed to blue light for more than 12 h, the cell viability began to decline significantly (*p* < 0.05). When blue-light exposure time exceeded 18 h, the viability of NIH/3T3 cells was lower than 50%, compared with the control group without blue-light irradiation treatment. As shown in Figure 5b, when the blue-light irradiation time was less than 6 h, the amount of ROS produced by the NIH/3T3 cells was similar to that of the control group without blue-light irradiation, and the cell viability was also similar. However, when NIH/3T3 cells were irradiated with blue light for 12 h, the amount of ROS produced was significantly increased, by 2.3-fold compared with the control group (*p* < 0.05), resulting in a significant decrease in cell viability. The amount of ROS produced by NIH/3T3 cells irradiated for 18 h was lower than that of those irradiated for 12 h. A possible reason for this is that the cell viability had been reduced to 50%, which was significantly lower than that of the group irradiated for 12 h. However, the amount of ROS produced for the 18 h group was significantly increased compared with the control group (*p* < 0.05). When the blue-light irradiation time was extended to 24 h, the viability of NIH/3T3 cells decreased to 35%, and the amount of ROS decreased due to the decrease in the number of surviving NIH/3T3 cells. The result shows that the maximum amount of ROS of NIH/3T3 cells was produced at 12 h of blue-light exposure time. Therefore, the following test of microalgal lutein against blue-light irradiated damage was chosen with an exposure time of 12 h.

### 3.6. Protection of NIH/3T3 Cells against Damage from Blue-Light Exposure by Lutein of Chlorella sp.

**Figure 6:**
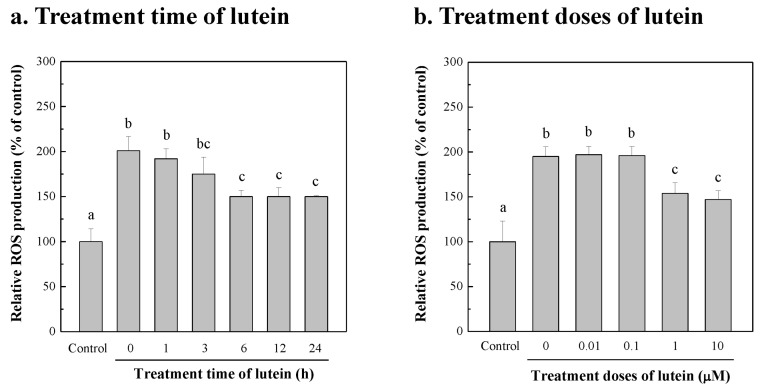
ROS production of NIH/3T3 cells treated with microalgal lutein for different time (**a**) and different doses (**b**) against the damage induced by blue-light irradiation. Before the cells were exposed to blue light with 300 μmol/m^2^/s of intensity for 12 h, NIH/3T3 cells were treated with 10 μM lutein for different time 0, 1, 3, 6, 12, and 24 h, and different doses of 0, 0.01, 0.1, 1, and 10 μM lutein for 6 h, individually. After exposure to blue light, the ROS production of NIH/3T3 cells was measured. The value of relative ROS production of NIH/3T3 cells without lutein supplement and no blue-light exposure was defined as control (C; 100%). All values were expressed as the mean ± SD. Data were compared with a one-way ANOVA test to evaluate the differences between multiple groups. Different letters indicate significant differences between groups.

To study whether lutein from *Chlorella* sp. produced using the fertilizer could reduce the ROS damage caused by blue-light irradiation, the different treatment time of microalgal lutein on NIH/3T3 cells from blue-light irradiation damage was explored. First, 10 μM microalgal lutein was added to treat NIH/3T3 cells for 0, 1, 3, 6, 12, and 24 h, followed by 12 h of blue-light irradiation to detect the amount of ROS produced by the NIH/3T3 cells. Figure 6a shows that after 0, 1, 3, 6, 12, and 24 h of treatment with 10 μM microalgal lutein, the relative ROS levels of NIH/3T3 cells were 201, 192, 175, 150, 150, and 150% of the control group, respectively. The control group (i.e., relative ROS level was 100%) was that without microalgal lutein supplement and blue-light irradiation. The results show that the NIH/3T3 cells exposed to blue light for 12 h produced two-fold the amount of ROS compared with the control group, and the amount of ROS could be reduced by increasing the microalgal lutein treatment time before blue-light irradiation for cell protection. Compared with a microalgal lutein treatment time of 0 h, when microalgal lutein was added to treat NIH/3T3 cells for more than 6 h, the amount of ROS produced by blue-light irradiation was significantly reduced from 201% to 150% of the control (*p* < 0.05). The result indicates that microalgal lutein can effectively reduce NIH/3T3 cell damage caused by blue-light exposure and achieve a protective effect.

The dose–effect response of microalgal lutein to protect the NIH/3T3 cells damaged by blue-light exposure was investigated. First, 0, 0.01, 0.1, 1, and 10 μM microalgal lutein was added to treat NIH/3T3 cells for 6 h, then blue-light irradiation was performed for 12 h to measure the amount of ROS produced by NIH/3T3 cells. As shown in Figure 6b, when 0, 0.01, 0.1, 1, and 10 μM microalgal lutein were added for 6 h treatment, the relative ROS levels of NIH/3T3 cells were 195, 197, 196, 154, and 147% of the control (without lutein supplement and blue-light irradiation), respectively. Low doses of lutein, less than 0.1 μM, did not reduce the NIH/3T3 cell damage resulting from blue-light exposure. However, when the dose of microalgal lutein was 1~10 μM, the amount of ROS produced by blue-light irradiation was significantly reduced (*p* < 0.05). The results mean that extracting lutein from *Chlorella* sp. cultured with the fertilizer reduced the ROS damage to NIH/3T3 cells caused by blue-light irradiation when a dose of ≥1 μM was used. The amount of ROS produced by blue-light exposure can be reduced by lutein treatment in Cristaldi et al. [34]. The lutein was purchased from Molekula Group (cat. no. 29291878). Compared with the control group cultured in the dark condition, the viability of human corneal epithelial cells decreased to 26% after blue-violet light irradiance, and the amount of ROS increased by 391% of control. Under a treatment of 100~250 μM lutein, the cell viability was increased by 85%, and the amount of ROS was reduced to only 126% of the control. The lutein standard can protect corneal epithelial cells from blue-light photo-oxidative and apoptotic damage. Similarly, the photo-protective effect of lutein was obtained from Sigma Chemical Co.; it also inhibited the production of ROS in UV-irradiated human keratinocyte (HaCaT) cells [82]. Taken together, lutein contributes to the reduction of ROS produced via photo-oxidation. Therefore, lutein produced by *Chlorella* sp. has great potential to develop diversified antioxidant products for nutritional supplements and skin protection.

## 4. Conclusions

This study demonstrated that the use of fertilizers can greatly reduce the cost of *Chlorella* sp. culture medium and further increase the lutein content of microalgal biomass using a cheap nitrogen source: urea supplementation. The cost of this culture medium per gram of microalgal biomass and per gram of lutein was reduced by 92% and 96%, respectively, compared with that of modified f/2 medium. A decrease in the media cost is more conducive to large-scale cultivation of *Chlorella* sp. In addition, the extracted lutein of *Chlorella* sp. can protect NIH/3T3 cells from ROS oxidation damage produced by blue-light irradiation. The produced microalgal lutein has great potential for developing high-value anti-photo-oxidation products. When the source and composition of the fertilizer have no biosafety concerns, the low-cost fertilizer medium can be used for large-scale cultivation to produce biomass of *Chlorella* sp. The functional component lutein from the produced microalgal biomass was extracted to protect cells from ROS oxidative damage caused by blue light irradiation, reduce the economic challenges of microalgae-based carbon fixation technology and microalgae-based biofuel production, and increase industrial practical application opportunities. In the future, low-cost fertilizer medium can be further investigated to determine its practical applications in large-scale cultivation of *Chlorella* sp. for CO_2_ fixation. It can also be coupled with the development of biofuels and high-value products through the production of microalgal biomass.

## Figures and Tables

**Table 1:** Comparison between the medium costs per liter of working volume, per gram of biomass production, and per gram of lutein production of *Chlorella* sp. for 6 days of cultivation.

Medium Costs	Modified f/2 Medium	20 g/L Fertilizer	20 g/L Fertilizer + 20 mM Urea
Cost per L working volume (US$)	0.276	0.011	0.023
Cost reduction (%) ^1^		96	92
Cost per g of biomass production (US$)	0.052	0.002	0.004
Cost reduction (%) ^2^		97	92
Cost per g of lutein production (US$)	16.35	0.382	0.665
Cost reduction (%) ^3^		98	96

^1^ The cost reduction per L working volume was calculated by comparison of the modified f/2 medium. ^2^ The cost reduction per g of biomass production was calculated by comparison of the modified f/2 medium. ^3^ The cost reduction per g of lutein production was calculated by comparison of the modified f/2 medium.

**Table 2:** Summary of the performance of growth and lutein production from *Chlorella* sp. for 6-day cultivation with nicotine, NaNO_3_, and urea addition in the fertilizer broth.

Extra Added Components	Concentration Added	Biomass Concentration (g/L)		Biomass Productivity ^1^ (g/L/d)		Max. Lutein Content ^2^ (mg/g)		Lutein Productivity (mg/L/d)	
Control ^3^		5.547 ± 0.464	^ad^	0.871 ± 0.079	^ad^	4.561 ± 0.327	^bc^	3.986 ± 0.564	^bd^
Nicotine	25 μM	5.263 ± 0.203	^ab^	0.826 ± 0.030	^ab^	5.425 ± 0.315	^ac^	4.472 ± 0.098	^abcd^
	50 μM	5.747 ± 0.100	^ad^	0.910 ± 0.019	^ad^	5.163 ± 0.250	^ac^	4.701 ± 0.304	^abcd^
	100 μM	5.791 ± 0.121	^ad^	0.917 ± 0.020	^ad^	4.992 ± 0.203	^abc^	4.581 ± 0.280	^abcd^
	200 μM	5.916 ± 0.163	^ad^	0.939 ± 0.026	^ad^	4.486 ± 0.284	^bc^	4.209 ± 0.255	^abcd^
NaNO_3_	5 mM	5.658 ± 0.140	^ad^	0.891 ± 0.023	^ad^	4.784 ± 0.347	^bc^	4.268 ± 0.420	^abcd^
	10 mM	5.710 ± 0.132	^ad^	0.901 ± 0.023	^ad^	5.396 ± 0.533	^ac^	4.867 ± 0.563	^cd^
	20 mM	5.694 ± 0.034	^ad^	0.901 ± 0.010	^ad^	4.732 ± 0.424	^bc^	4.259 ± 0.339	^abcd^
	30 mM	6.165 ± 0.177	^d^	0.977 ± 0.023	^d^	4.326 ± 0.366	^bc^	4.226 ± 0.326	^abcd^
	60 mM	5.456 ± 0.075	^acd^	0.860 ± 0.011	^acd^	4.022 ± 0.428	^b^	3.458 ± 0.413	^b^
Urea	5 mM	5.822 ± 0.116	^ad^	0.915 ± 0.023	^ad^	4.322 ± 0.443	^bc^	3.955 ± 0.459	^bd^
	10 mM	5.863 ± 0.222	^ad^	0.921 ± 0.030	^ad^	5.114 ± 0.286	^abc^	4.712 ± 0.315	^abcd^
	20 mM	5.648 ± 0.258	^ad^	0.890 ± 0.036	^ad^	6.031 ± 0.258	^a^	5.363 ± 0.231	^ac^
	30 mM	4.667 ± 0.140	^bc^	0.725 ± 0.020	^bc^	4.990 ± 0.456	^abc^	3.611 ± 0.225	^bd^
	60 mM	4.606 ± 0.272	^b^	0.714 ± 0.053	^b^	4.776 ± 0.362	^bc^	3.423 ± 0.497	^b^

^1^ Biomass productivity was calculated from biomass concentration in a 6-day culture. ^2^ Maximum lutein content was obtained after 6 days of cultivation. ^3^ The control group was 20 g/L fertilizer as the culture broth. Data were compared with a one-way ANOVA test to evaluate the differences between multiple groups on biomass concentration, biomass productivity, lutein content, lutein productivity, and different letters (^a, b, c^ and ^d^) indicate significant differences between groups (*p* < 0.05) in the same column based on the Bonferroni test.

## Data Availability

The data presented in this study are available upon request from the corresponding author.
